# Tackling healthcare-associated infections in Brazilian intensive care units: we need more than collaboration

**DOI:** 10.5935/0103-507X.2022editorial-en

**Published:** 2022

**Authors:** Renato Daltro de Oliveira, Pedro Fortes Osório Bustamante, Bruno Adler Maccagnan Pinheiro Besen

**Affiliations:** 1 Intensive Care Unit, A.C. Camargo Cancer Center - São Paulo (SP), Brazil.; 2 Trauma and Acute Care Surgery Intensive Care Unit, Hospital das Clínicas, Faculdade de Medicina, Universidade de São Paulo - São Paulo (SP), Brazil.; 3 Medical Intensive Care Unit, Hospital das Clínicas, Faculdade de Medicina, Universidade de São Paulo - São Paulo (SP), Brazil.

Healthcare-associated infections (HAIs) and antimicrobial resistance (AMRs) demand a global effort for their containment.^(1)^ Healthcare-associated infections have an estimated prevalence between 7 and 10% worldwide, whereas the incidence is greater than 15% in developing countries. This difference is more remarkable when comparing the incidence of HAIs in intensive care units (ICUs) with estimated values of 47.9 per 1,000 patient days in developing countries and 13.9 per 1,000 patient days in the United States.^(2)^

The impact of HAIs is wide in scope. Patients diagnosed with HAIs have a longer length of stay (LOS) and higher mortality, especially when infections are associated with antimicrobial resistance.^(3,4)^ Cassini et al. demonstrated that greater than 670,000 patients died from AMR in 2015, and 63.5% of cases were healthcare associated. These infections were also associated with a significant number of disability-adjusted life-years.^(5)^ Approximately US$15 billion were spent in 2016 on HAIs, and approximately €8,500 to 34,000 more were spent per infection due to longer LOS and additional treatment, as estimated by the *Organização para a Cooperação e Desenvolvimento Econômico* (OECD).^(6,7)^

Efforts have been made to reduce the incidence of HAIs and their downstream effects. Infection prevention and control (IPC) interventions, such as hand hygiene and perioperative prophylaxis, can successfully mitigate HAI occurrence.^(8,9)^ However, IPC programs are limited and unevenly distributed worldwide. Data from a system to monitor the status of progress toward the implementation of the AMR global action plan (the Tripartite Antimicrobial Resistance Country Self-assessment Survey - TrACSS) have shown that approximately 34% of countries reported having an IPC program implemented nationwide.^(1)^

In Brazil, data regarding HAIs are sparse. In this issue of *Revista Brasileira de Terapia Intensiva*, Melo et al. report the perceived success factors of a collaborative quality improvement project conducted from 2018 to 2019 in five ICUs from hospitals in the metropolitan region of Recife.^(10)^ Some ICUs started from a high baseline level in ventilator-associated pneumonia (VAP) rates, with median VAP rates in the 20’s range, with an observed reduction after the collaborative effort. For catheter-related bloodstream infections (BSI), median rates ranged from 5 to 10, but no reductions were observed. For urinary catheter-associated urinary tract infections, a marked reduction could be observed in two hospitals with high baseline rates. The collaborative effort led to reductions in HAIs but not to levels achievable, as described in the literature, such as the near-zero levels potentially obtainable for BSIs. The authors report that the factors associated with success in the most successful hospital were the full engagement of ICU staff, including committed ICU medical and nurse directors leadership, and active participation of the multiprofessional team along with an ICU consultant and chief nurse who were responsible for daily rounds. The ICU nurses engaged in Plan-Do-Study-Act (PDSA) cycles and educational activities. In contrast, hospitals that could not demonstrate reductions did not fully engage the leadership and the medical team due to perceived work overload or other issues.

These results are significant because they show the potential to improve HAI rates in Brazilian hospitals. On the other hand, these results are alarming due to the high rates of HAIs and because they are still far from being near optimal, which begs the question: how can we achieve high-quality critical care in Brazil (which includes low levels of HAIs)?

First, to achieve better results for HAI and other outcomes in critically ill patients, we need to strive for better nursing and multiprofessional team staffing in Brazil.^(11)^ Unfortunately, current Brazilian regulations are soft, and this will be a continuous barrier to improvement in best practices for preventing HAIs or other outcomes. We need to ensure that proper staffing is secured for the provision of safe critical care practices through proper regulation and regular auditing by regulatory authorities.

However, improving staffing is not sufficient. We need to train the next generation of intensivists (physicians, nurses, pharmacists, physical therapists and other team members) to the tenets of modern critical care. Risks and benefits need to be balanced to avoid the excesses of critical care and expose patients to the minimum invasiveness necessary for recovery.^(12)^ Continuous quality improvement must be part of ICU care through data-driven^(13)^ decisions and should not be implemented in a top-down manner. A bottom-up strategy where the team embraces this as their responsibility is the key to better outcomes. External views may help expose problems and identify potential solutions, but the results ultimately depend on team engagement and accountability for their results.

Third, the structure and provision of material and equipment necessary for proper care are essential. Administrators and stakeholders not involved in direct care need to take their responsibility seriously and deliver what is necessary. In public institutions, cuff meters commonly are not replaced when damaged or alcohol swabs to cleanse catheter connections become unavailable, exposing patients to avoidable harm, professionals to moral distress and burnout^(14)^ and our healthcare system to unnecessarily increased costs and inefficiency.

In addition to these needs, there has been increasing interest in tele-ICU coverage, another proposed solution to improve Brazilian ICU care, and results from clinical trials are eagerly awaited.^(15)^ Although tele-ICU coverage may provide needed expertise at the point of care in regions where intensivists are unavailable, it cannot be considered the solution to all our problems, especially as we pave our way out of the COVID-19 pandemic. To move forward and improve Brazilian ICU care, we still need to aim at the four fundamental aspects for the provision of high-quality critical care:

- Better staffing ratios with critical care trained healthcare professionals at the point of care.
- Data-driven ICU management and continuous efforts for quality improvement.
- Well-designed, evidence-based processes of care.
- Provision of a reliable structure to ensure that processes of care can be delivered without shortcomings.

This requires regulation, auditing and investment in Brazilian ICUs. Nationwide collaborative initiatives are all welcome to improve care and reduce the burden of HAIs in Brazilian ICUs, but ultimately we need to empower local teams and give them the tools for improvement if we wish to achieve the best possible outcomes.

## References

[1] Balakrishnan VS (2022). WHO’s first global infection prevention and control
report. Lancet Infect Dis.

[2] Allegranzi B, Bagheri Nejad S, Combescure C, Graafmans W, Attar H, Donaldson L (2011). Burden of endemic health-care-associated infection in developing
countries: systematic review and meta-analysis. Lancet.

[3] Zhou Q, Fan L, Lai X, Tan L, Zhang X (2019). Estimating extra length of stay and risk factors of mortality
attributable to healthcare-associated infection at a Chinese university
hospital: a multi-state model. BMC Infect Dis.

[4] Barrasa-Villar JI, Aibar-Remón C, Prieto-Andrés P, Mareca-Doñate R, Moliner-Lahoz J (2017). Impact on morbidity, mortality, and length of stay of
hospital-acquired infections by resistant microorganisms. Clin Infect Dis.

[5] Cassini A, Högberg LD, Plachouras D, Quattrocchi A, Hoxha A, Simonsen GS, Colomb-Cotinat M, Kretzschmar ME, Devleesschauwer B, Cecchini M, Ouakrim DA, Oliveira TC, Struelens MJ, Suetens C, Monnet DL, Burden of AMR Collaborative Group (2019). Attributable deaths and disability-adjusted life-years caused by
infections with antibiotic-resistant bacteria in the EU and the European
Economic Area in 2015: a population-level modelling analysis. Lancet Infect Dis.

[6] Forrester JD, Maggio PM, Tennakoon L (2022). Cost of health care-associated infections in the United
States. J Patient Saf.

[7] Organization for Economic Cooperation and Development
(OECD)/European Union (2018). State of health in the EU cycle.

[8] Allegranzi B, Pittet D (2009). Role of hand hygiene in healthcare-associated infection
prevention. J Hosp Infect.

[9] Leaper DJ, Edmiston CE (2017). World Health Organization: global guidelines for the prevention
of surgical site infection. J Hosp Infect.

[10] Melo LS, Estevão TM, Chaves JS, Vieira JM, Siqueira MM, Alcoforado IL (2022). Success factors of a collaborative project to reduce
healthcare-associated infections in intensive care units in Northeastern
Brazil. Rev Bras Ter Intensiva.

[11] Zampieri FG, Salluh JI, Azevedo LC, Kahn JM, Damiani LP, Borges LP, Viana WN, Costa R, Corrêa TD, Araya DE, Maia MO, Ferez MA, Carvalho AG, Knibel MF, Melo UO, Santino MS, Lisboa T, Caser EB, Besen BA, Bozza FA, Angus DC, Soares M, ORCHESTRA Study Investigators (2019). ICU staffing feature phenotypes and their relationship with
patients’ outcomes: an unsupervised machine learning
analysis. Intensive Care Med.

[12] Lobo SM, Mendes CL, Rezende E (2020). Choosing Wisely in intensive care medicine. Rev Bras Ter Intensiva.

[13] Zampieri FG, Soares M, Salluh JI (2020). How to evaluate intensive care unit performance during the
COVID-19 pandemic. Rev Bras Ter Intensiva.

[14] Castro CS, Timenetsky KT, Katz M, Corrêa TD, Felicio AC, Moriyama T (2020). Burnout syndrome and engagement among critical care providers: a
cross-sectional study. Rev Bras Ter Intensiva.

[15] Ranzani O, Pereira AJ, Santos MC, Corrêa TD, Ferraz LJ, Cordioli E (2022). Statistical analysis of a cluster-randomized clinical trial on
adult general intensive care units in Brazil: TELE-critical care verSus
usual Care On ICU PErformance (TELESCOPE) trial. Rev Bras Ter Intensiva.

